# Effect of Mat Pilates Training on Blood Pressure, Inflammatory, and Oxidative Profiles in Hypertensive Elderly

**DOI:** 10.3390/sports12050120

**Published:** 2024-04-28

**Authors:** Chutima Woramontri, Rungchai Chaunchaiyakul, Ai-Lun Yang, Yi-Yuan Lin, Kunanya Masodsai

**Affiliations:** 1 Exercise Physiology in Special Population Research Unit, Faculty of Sports Science, Chulalongkorn University, Bangkok 10330, Thailand; 6470009639@student.chula.ac.th; 2 College of Sports Science and Technology, Mahidol University, Nakhonpathom 73170, Thailand; rungchai.chy@mahidol.ac.th; 3 Institute of Sports Sciences, University of Taipei, Taipei 11153, Taiwan; alyang@utaipei.edu.tw; 4 Department of Exercise and Health Science, National Taipei University of Nursing and Health Sciences, Taipei 11219, Taiwan; yiyuanlin@ntunhs.edu.tw

**Keywords:** Pilates, blood pressure, hypertension, anti-inflammation, anti-oxidation, cardiovascular function

## Abstract

To determine the effects of mat Pilates training on blood pressure, inflammatory, and antioxidative markers in hypertensive elderly people, 34 hypertensive subjects aged 60–75 years were randomly divided into a control group (CON; *n* = 17) and a mat Pilates training group (MP; *n* = 17). The CON participants conducted normal daily activities and participated in neither organized exercises nor sports training, while those in the MP group received mat Pilates training for 60 min three times/week for 12 weeks. Parameters including blood pressure, cardiovascular function, nitric oxide (NO), tumor necrotic factor-alpha (TNF-α), superoxide dismutase (SOD), and malonaldehyde (MDA) were collected at baseline and the end of 12 weeks. The MP group had significantly decreased blood pressure, improved cardiovascular variables, decreased MDA and TNF-α, and increased NO and SOD compared with the CON group and the pre-training period (*p* < 0.05). In conclusion, these findings demonstrate the positive effects of 12 weeks of mat Pilates training in terms of reducing blood pressure and increasing blood flow related to improvements in anti-inflammatory and antioxidative markers in hypertensive elderly people. Mat Pilates training might be integrated as an alternative therapeutic exercise modality in clinical practice for hypertensive elderly individuals.

## 1. Introduction

Hypertension is a significant risk factor in various cardiovascular conditions, including coronary heart disease, stroke, and heart failure. The World Health Organization estimates that as many as 1.4 billion individuals aged 30–79 years worldwide may be affected by hypertension, and this number is projected to rise to approximately 1.56 billion in 2025 [1]. Hypertension in the elderly is one of the most significant age-related deteriorations and a major global health public challenge, with increasing numbers worldwide [2]. It is characterized by persistent blood pressure (BP) elevation, thereby amplifying the risk for cerebral, cardiac, and renal abnormalities [3]. Regulating blood pressure involves coordinating multiple organs and systems within the body. The primary risk factor for the development of hypertension is the heart’s efforts to compensate for high systemic vascular resistance (SVR) within the circulatory system [4]. Aging has a significant influence on the decline of cardiovascular function and may subsequently contribute to manifestations of cardiovascular diseases (CVDs) such as hypertension [5]. Elderly individuals often experience high SVR, which is a significant pathophysiological characteristic. As a result, the eventual outcome of this condition—high blood pressure—is likely to be evident [6]. Moreover, arteriosclerotic changes, either from aging or hypertension, arise because of impaired vascular compliance [7] resulting from endothelial dysfunction and reduced levels of vasodilator substances, such as nitric oxide (NO). Endothelial dysfunction primarily stems from a decrease in the availability of NO. This can manifest through various markers, such as diminished endothelium-dependent vasorelaxation, the heightened expression of adhesion molecules and inflammatory cytokines, the induction of reactive oxygen species (ROS), impaired fibrinolytic activity; elevated turnover rates, and excessive growth factor production [8,9]. In addition to these factors, other inflammatory and oxidative markers contribute to this condition, such as tumor necrotic factor-alpha (TNF-α) and malondialdehyde (MDA) [10]. The above oxidative and inflammatory markers have been studied in an animal model [10]. The impacts of aging and hypertension on cardiovascular dysfunction are significant, and their intricate interplay has been scientifically explored and confirmed. One of our studies using an animal model revealed that aging appears to exacerbate insulin and insulin-like growth factor-1 (IGF-1)-mediated endothelial dysfunction, particularly through impairing the phosphoinositide 3-kinase (PI3K)–nitric oxide synthase (NOS)–NO pathway in spontaneously hypertensive rats (SHRs). This effect is further compounded by decreased nitric oxide production and an imbalance between oxidative and antioxidant activities in hypertension [5].

Exercise is an effective non-pharmacological approach to the prevention and treatment of cardiovascular disease and hypertension [11]. Previous systematic reviews have demonstrated that various types of exercise training, including endurance and resistance training, can significantly reduce systolic (SBP) and diastolic blood pressure (DBP) in healthy adults and those with hypertension [12]. This appears to be true even in individuals with low responsiveness to medical treatment, who can experience a reduction in blood pressure through physical exercise training [13]. Aerobic exercise is primarily recommended for blood pressure reduction [14]. The current exercise prescription for treating hypertension is an aerobic training mode of 20–60 min/day, 3–5 days/week, at 40–70% of maximum oxygen uptake (VO_2max_) [15]. Other modes of antihypertensive exercise training explored in previous studies include walking [16], water-based exercise [17], handgrip exercise [18], and leg and arm exercises [19]. Isometric exercise and high-intensity interval training (HIIT) [20] have been reported to have different levels of effectiveness in lowering BP [21]. Moreover, previous evidence suggests that the reduction in blood pressure induced by exercise is associated with an improvement in endothelial function, as evaluated through endothelium-dependent vasodilation [12], possibly via a decrease in inflammation and an increase in antioxidant capacity [22]. A previous study on middle-aged hypertensive patients who underwent 12 weeks of supervised aerobic training indicated a reduction in 24 h blood pressure alongside reduced inflammatory responses, including lower levels of C-reactive protein (CRP) and monocyte chemoattractant protein-1 (MCP-1) [23].

Recent reviews have shown that physical activity is widely recommended as one of the primary ways to promote positive psychophysiological health in individuals [24]. Despite the indisputable positive benefits of exercise in lowering BP, adherence to exercise and maintenance rates are surprisingly low [25]. Pilates is an easy exercise that focuses on improving strength, coordination, balance, and postural control [26] through a targeted series of full-body movements while using one’s own body weight as resistance, and it has become more popular among older groups [27]. The effects of Pilates training on health benefits can be demonstrated in different forms: tele-Pilates [28], combined with yoga [29], and mat Pilates (MP) [30]. The mat Pilates program incorporates core strengthening, symmetrical movements of the legs and arms, and breathing control with body awareness [31]. For the elderly, mat Pilates is considered a low-impact and safe exercise and has minimal expense [32]. The effects of Pilates have been explored in certain aspects among hypertensive people, showing changes in heart rate [33], quality of life, and body composition [34]. A recent review reported Pilates intervention in a hypertensive population with a small sample size and controversial outcomes [35]. Although some previous reports have demonstrated that Pilates can decrease blood pressure in hypertensive participants, the aforementioned study was limited to adult women under the effects of antihypertensive medications [36], and the mechanisms behind the process were limited, with both acute [37] and minor training alterations [34].

Research on the impact of Pilates training on blood pressure is still inconclusive. Initial investigations among normotensive populations have indicated that Pilates does not significantly affect blood pressure. However, more recent studies have reported positive and significant changes in both SBP and DBP in hypertensive adults. However, studies involving hypertensive patients have yielded conflicting results [35]. Moreover, hypertensive elderly people exhibit severe, pathology-including biomarkers of oxidative stress and inflammation. Therefore, mat Pilates training may decrease blood pressure and regulate oxidative stress and inflammation; thus, it may be useful as an alternative non-pharmacological intervention. Given the inconclusive results of previous studies on mat Pilates and a lack of changes in blood biochemical indicators, the present study aimed to investigate the effects of this form of training on BP and markers of inflammation and oxidative stress in hypertensive elderly people using a human randomized controlled trial design.

## 2. Materials and Methods

### 2.1. Participants

The required sample size was calculated with the G*power program using data from a previous study, with test power at 0.8 and a portable error of 0.05. Thirty-four volunteers with an age range of 60–75 years were recruited through social networks and flyers. The inclusion criteria were those who had stage 1 hypertension (systolic and diastolic blood pressure, SBP and DBP: 140–159 and 90–99 mmHg, respectively), were free of previously diagnosed psychologically related hypertension, and had irregularly participated in any physical training (less than 2 times/week) over the last six months. The exclusion criteria were those who had musculoskeletal problems, recent drug treatment changes, exertion angina, heart palpitations, or any cardiovascular events or symptoms that limited the participant from continuing the exercise training program. This randomized (computer-based) controlled clinical trial was carried out and conducted at the Exercise Physiology in Special Populations Unit. Participants were then divided into two groups: the control group (CON; no regular physical exercise training more than 2 times/week throughout the experimental period) and the mat Pilates training group (MP; structurally designed mat Pilates 3 times/week). All participants were informed of the study’s objective, procedures, benefits, and potential risks before participation. During a 12-week training period, all participants completed the informed consent forms. The CON participants were asked to maintain normal daily routine activities and regular meals during the twelve-week study period, and they were frequently tracked via social media (the Line application). Approval for the project was obtained from the Ethics Committee on Human Experiments, Chulalongkorn University (650176).

### 2.2. Mat Pilates Training Program

The mat Pilates training session consisted of 60 min divided into 10 min of warming up and stretching, 40 min of MP exercise, and 10 min of stretching and cooling down. For safety purposes, this study ran 16 mat Pilates training exercises (Table 1) in a supine position based on the 5 main principles of Pilates: head and cervical placement; shoulder blade movement and stability; breathing control; ribcage placement; and pelvic placement. To maintain all participants’ adherence, the MP training classes were regularly conducted by an internationally licensed Pilates instructor (CW) and two assistants, who were both sports scientists. A 1-week familiarization with Pilates movements was applied to all participants in the MP group. Participants performed 3 rounds of circuit training of 16 MP exercises of 10–15 reps/set, 3 sets/day, and 3 days/week for a total of 12 weeks. According to the principle of progressive training, these MP interventions were separated into 3 periods of 40%, 50%, and 60% heart rate reserve (HRR). HRR was calculated using the following equation:

HRR = HRmax − HRrest,(1)

where HRmax is the maximal heart rate from Tanaka’s study [38], and HRrest is the resting heart rate. During Pilates sessions, chest belt BP monitors (Polar H-10, Polar Electro, Kempele, Finland) and synchronized watches were used for HR monitoring. Reps and sets were estimated weekly to identify the individual’s intensity. Before commencing MP, a subject was allowed to sit quietly until stable BP and HR were achieved. Subjective evaluations were also conducted using the rating of perceived exertion (RPE, 6–20 scale). On non-training days, MP participants were also asked to maintain normal daily activity and regular diets, and they were frequently tracked via the Line application. 

### 2.3. Physiological Variables and Measurements

Anthropometric variables, including body weight, height, body mass index (BMI), waist/hip ratio, body fat, and lean body mass, were collected using a bioelectrical impedance analyzer (BIA, IOI, Jawon Medical, Seoul, Republic of Korea) [39]. Cardiac variables were determined using non-invasive impedance cardiography (Physioflow PF07 Enduro, Manatec Biomedical, Paris, France), according to the standard methods from a previous study [40]. These included heart rate (HR), SBP, DBP, mean arterial BP (MABP), pulse pressure (PP), cardiac output (CO), stroke volume (SV), and SVR. Vascular wall thickness and intima–media thickness (IMT) were determined using ultrasound-image techniques (EPIQ5, Philips Healthcare, Andover, MA, USA) with an analysis program (Vascular Research Tools, Medical Imaging Applications LLC, Coralville, IA, USA). Moreover, arterial stiffness as measured by brachial–ankle pulse wave velocity (baPWV) was detected and analyzed using a non-invasive vascular screening device (Colin VP–1000 plus, Omron, Ukyo-ku, Kyoto, Japan). The low frequency-to-high frequency ratio (LF/HF ratio) of heart rate variability (HRV) was determined using a telemetry heart rate monitor (Polar H10, Poland) and the HRVelite application [41].

### 2.4. Blood Collection and Biochemical Analysis

In this study, blood samples were obtained from antecubital veins by licensed nurses and left for clotting for 30 min at room temperature. After centrifugation at 2000× *g* and 4 °C for 15 min, the serum was separated from the whole blood contents and stored at −80 °C in a refrigerator for later biochemical analysis. Anti-inflammatory and antioxidative markers and nitric oxide concentrations were carefully analyzed using an enzyme-linked immunosorbent assay (ELISA). Serum MDA concentration, an index of the lipid peroxidation marker, was determined using a thiobarbituric acid reactive substances (TBARS) assay kit (Abcam, Cambridge, UK), prepared according to the manufacturer’s protocol, and mixed with the serum samples to generate MDA-thiobarbituric acid (TBA) adducts under high temperatures (90–100 °C) and acidic conditions. After completing the reactions, samples were measured with colorimetry at 540 nm with a microplate reader (SpectraMax iD3, Molecular Devices, CA, USA). The concentration was expressed in μM in the serum samples. Serum superoxide dismutase (SOD) activity was determined using a SOD assay kit (Abcam, Cambridge, UK). The assay used a tetrazolium salt to detect superoxide radicals generated by xanthine oxidase and hypoxanthine. One unit of SOD was defined as the amount of enzyme needed to exhibit 50% dismutation of the superoxide radicals. The absorbance was read at 450 nm by a microplate reader (SpectraMax iD3, Molecular Devices, CA, USA), and the activity was expressed in U/mL in the serum samples. Serum tumor necrotic factor-alpha was analyzed using a TNF-α assay kit (Abcam, Cambridge, UK). The TNF-α antibody was added to initiate a reaction, followed by a TMB solution after washing. Then, the absorbance was read at 450 nm with a microplate reader (SpectraMax iD3, Molecular Devices, CA, USA) after adding the stop solution. Moreover, the serum nitric oxide concentration was analyzed as the level of nitrate/nitrite in the serum using its assay kit (Abcam, Cambridge, UK). Briefly, the nitrate reductase and enzyme cofactor were added to the wells. After 1 h of incubation at room temperature, the Griess reagents were added before reading at 540 nm with a microplate reader (SpectraMax iD3, Molecular Devices, CA, USA). The result is expressed in mM units. 

All physiological and biochemical variables were carefully determined at rest as baseline (pre-test) and at the end of 12 weeks (post-test).

### 2.5. Statistical Analysis

All data are presented as the means ± standard deviation (SD). The SPSS software (Version 21, IBM, Armonk, NY, USA) was used to perform all statistical analyses. The normality of the data was confirmed with the Shapiro–Wilk test. Independent sample *t*-tests were used to compare groups at baseline. Repeated measures ANOVAs were performed for each outcome variable to investigate differences between groups (MP vs. CON) over time (baseline vs. after 12 weeks of intervention) with the Bonferroni test. A *p*-value less than 0.05 was considered statistically significant.

## 3. Results

### 3.1. General Characteristics

This study recruited equal numbers to the CON (*n* = 17) and MP (*n* = 17) groups, with no significant difference (*p* > 0.05) in mean ages between CON (65.35 + 3.94 years) and MP (65.18 + 3.83 years). The average height of the CON and MP groups revealed no significant difference at 159.30 ± 8.80 cm and 157.90 ± 8.83 cm (*p* = 0.387, η^2^ = 0.108), respectively. MP training took about 1 h for all 16 MP exercises, with no signs or symptoms of abnormality. All members of the MP group participated in more than 80% of the mat Pilates program. Anthropometric data (Table 2) on body weights, body mass index, waist/hip ratio, body fat, and lean body mass were not significantly different at the beginning of the study (*p* > 0.05, pre-test to post-test). By the end of the 12 weeks, there were significant reductions in body fat (*p* < 0.05) and significantly increased lean body mass in the MP group compared with baseline and the CON group (*p* < 0.05).

### 3.2. Cardiovascular Function

Table 3 reveals changes in cardiovascular functions. None of the resting cardiovascular variables (pre-test to pre-test) showed any significant differences between the groups (*p* > 0.05). However, there were significant improvements in SBP, MABP, PP, SV, and SVR within a group (pre-test to post-test) (*p* < 0.05) and between (post-test to post-test) (*p* < 0.05) groups. IMT showed a significant difference between groups (*p* < 0.05), while the LF/HF ratio showed a significant difference compared with baseline only (*p* < 0.05). The present study showed that 12-week MP training decreased SBP by about 16 mmHg.

### 3.3. Anti-Inflammatory and Antioxidative Markers

**Figure 1:**
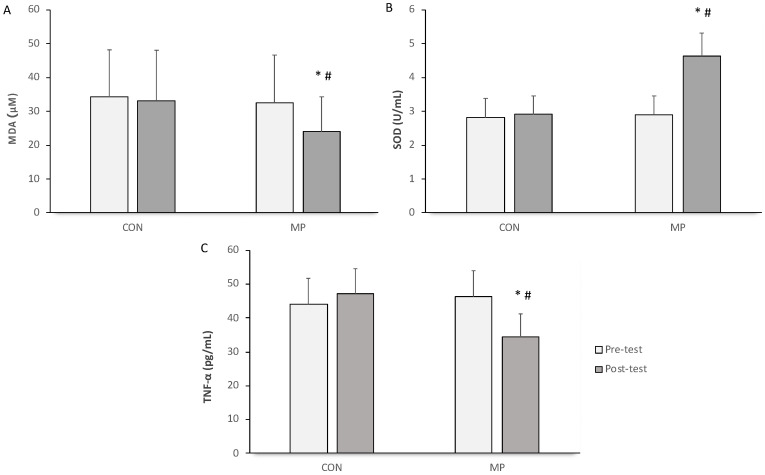
Changes in serum malonaldehyde (MDA; (**A**)), superoxide dismutase (SOD; (**B**)), and tumor necrotic factor-alpha (TNF-α; (**C**)) concentration in control (CON) and mat Pilates (MP) groups during pre- (white square) and post-training (dark square) tests. *, *p* < 0.05, significant difference within a group; #, *p* < 0.05, significant difference between groups at the same phase.

There were no significant differences in MDA, SOD, and TNF-α levels between the pre- and post-tests in the CON group (Figure 1) while, in the MP group, TNF-α and MDA levels showed significant reductions (*p* < 0.05) with a significant elevation in SOD levels (*p* < 0.05) after 12 weeks of MP training.

### 3.4. Serum Nitric Oxide Level

Nitric oxide levels are represented by their nitrate/nitrite byproduct concentrations in blood. There was no significant change in serum nitrate/nitrite between the pre-test and post-test in the CON group. On the other hand, the MP group showed higher serum nitrate/nitrite concentrations within the group (pre-test to post-test *p* < 0.05) and between groups (post-test to post-test, with CON, *p* < 0.05).

## 4. Discussion

The results indicate the effectiveness of twelve weeks of mat Pilates training in reducing blood pressure, improving cardiovascular functions, and enhancing anti-inflammatory and antioxidative markers in hypertensive elderly people. In addition, this study also shows that mat Pilates training for 12 weeks facilitates vasodilation via nitric oxide concentration in serum. The challenge with hypertension in the elderly lies in its asymptomatic nature, making it difficult for individuals to realize their condition and increasing the risk of having an uncontrolled condition, which can precipitate various medical situations. Thus, the precise medical prescriptions for hypertensive treatments vary among clinicians because of differential guideline recommendations with variable side effects [42]. The present investigation of blood markers provides a clearer understanding of mat Pilates training as an antihypertensive management model. The reduction in oxidative and inflammatory markers in parallel with increasing SOD and NO in the MP group revealed that MP training would not aggravate the risk of cell damage.

MP training in this study was designed to simulate aerobic exercise, which involves repeated rhythmic contractions of large skeletal and core muscle groups performed for a long period, at least 60 min. A period of regular MP training lowered SBP and DBP and enhanced CO and SV (Table 3), which, in turn, provided a greater oxygen supply to the active muscles of the limbs and core. A previous in-depth investigation [43] on endurance training revealed similar changes in cardiovascular functions. The MP intervention in the present study demonstrates both antihypertension and a reduction in cardiac autoregulation.

The mat Pilates training in this study also exhibits beneficial compensations both within (cardiac contractility, SV and CO) and without the cardiac chamber (systemic vascular resistance, SVR). Reductions in both systolic and diastolic blood pressure from aerobic training have been noted in previous studies, in parallel with an increased left ventricular (LV) ejection fraction, decreased end-diastolic pressure, improved vascular function, and possibly enhanced cardiac angiogenesis and cardiac muscle mass [44,45,46]. Changes in the flow rates of both the right and left baPWVs (Table 3) represent enhanced vascular function, in particular, vasodilation. Thus, MP training induces physiological adaptations that reduce cardiac workload via diminutions of pressure loading, as well as a reduction in SVR.

**Figure 2:**
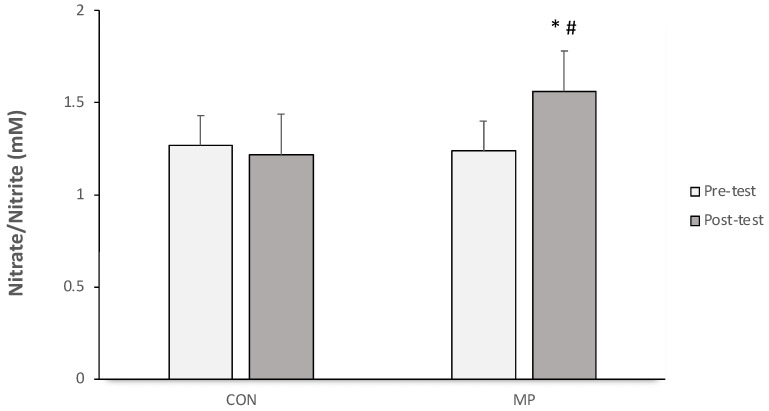
Changes in serum nitrate/nitrite concentration in CON and MP groups during pre- (white square) and post-training (dark square) periods. *, *p* < 0.05, significant difference within a group; #, *p* < 0.05, significant difference between groups at the same phase.

TNF-α is a pluripotent cytokine produced by macrophages and adipocytes [47]. It represents local inflammatory responses mediated by specific membrane receptors [48,49]. The present study shows a lower serum TNF-α value in the MP group (Figure 2), in parallel with a previous study [50]. This indicated that regular Pilates exercise at moderate-to-high intensity induces anti-inflammatory effects with elevated levels of anti-inflammatory cytokines and suppressed proinflammatory makers, as represented by TNF-α. One study showed that aerobic exercise exhibits an anti-inflammatory effect and can be an appropriate training protocol that can reduce plasma TNF-α concentrations even in a healthy state [50]. However, another study demonstrated TNF-alpha downregulation in diabetic patients after exercise training [51]. In an animal model, the health benefits of exercise training can be demonstrated; it can enhance the ability of isolated adipocytes to secrete TNF-α to reduce soluble tumor necrosis factor receptor 1 (sTNFR1; a member of the tumor necrosis factor receptor superfamily) receptor secretion, which plays a major role in apoptosis, cell survival, differentiation, and inflammation [52].

Aerobic exercise via MP training results in repeated and prolonged exposure to shear stresses on the arterial wall; then, a cascade mechanism takes place, improving nitric oxide bioavailability (Figure 2), a key mediator for endothelial function. In addition, regular aerobic training has been shown to attenuate endothelial dysfunction in the aged population [53]. A study of the antihypertensive effects of regular exercise training revealed a crucial mechanism in that physical activity improves endothelium-dependent vasodilation in the hypertensive population, although this depends on the type of exercise (aerobic, resistance, or concurrent training) [54]. The present study confirms that NO is higher following mat Pilates training, and this evidence should facilitate the prescription of MP as a therapeutic exercise for hypertensive elderly people. 

There is growing evidence that increased oxidative stress and associated oxidative damage are mediators of vascular injury in cardiovascular pathologies, including hypertension and atherosclerosis [55]. The balance between oxidative stress and antioxidative levels must be tightly maintained to protect the cells from damage [56]. In hypertension, especially among the elderly, an imbalance in antioxidant status exists and can subsequently impair nitric oxide availability [5]. On the other hand, evidence suggests that exercise training can reduce blood pressure and improve endothelial function [57,58], potentially through mechanisms such as decreased inflammatory cytokines and increased antioxidant capacity [22]. A previous study reported that moderate-intensity exercise tends to decrease the indices of oxidative stress, such as 8-hydroxy-2′-deoxyguanosine (8-OHdG) and malondialdehyde-modified low-density lipoprotein (MDA-LDL) [59]. Moreover, aerobic exercise training also increases blood antioxidant levels in animal models [60]. Our results demonstrate that twelve-week mat Pilates training relieves systemic inflammation and oxidative stress in hypertensive conditions by downregulating MDA and upregulating SOD levels (Figure 1). In other words, mat Pilates training regulates the balance between ROS and their scavengers, resulting in decreased oxidative stress in hypertensive elderly people. One of the suggested mechanisms is the redox regulation of exercise-induced blood flow. Exercise induces blood flow and leads to increased vascular shear stress and then induces mitochondrial superoxide (mtO_2_^•−^) production via nicotinamide adenine dinucleotide phosphate (NADPH) oxidase and c-Jun NH2-terminal kinase (JNK-1 and JNK-2) signaling [61]. Superoxide dismutase plays a vital role in protecting cells against the damaging effects of the superoxide radical (O_2_^•−^), as it acts alongside other antioxidative enzymatic networks to eliminate harmful free radicals [62]. One study showed that aerobic exercise can induce endogenous antioxidants by increasing several differentially expressed proteins in a training group [63]. Thus, the results of this study confirmed the antioxidant effect of our mat Pilates training, which was designed based on the principle of aerobic training to provide the most benefit in treating hypertension.

However, other health conditions require further investigation to determine the optimal therapeutic effect of mat Pilates. Furthermore, some limitations in this study are as follows: (a) MP exercises used in this study were very specific to hypertension stage 1; (b) the time–dose effectiveness of MP before 12 weeks needs to be explored.

## 5. Conclusions

Mat Pilates training for 12 weeks led to multifaceted impacts, reducing blood pressure, inflammatory and oxidative markers, and intima–media wall thickness and improving antioxidative markers and cardiovascular functions. Thus, it emerges as a promising intervention for hypertensive elderly people, demonstrating reductions in cardiac loading and vascular compensations. The success of mat Pilates training in the present study offers significant potential in realizing its health benefits. 

## Figures and Tables

**Table 1:** Mat Pilates program based on five main principles.

Mat Pilates	Principles
Half rollback	Head, cervical, and ribcage placement Shoulder blade stability
Oblique rollback	Head, cervical, and ribcage placement Shoulder blade stability
Hundred prep	Breathing control Ribcage placement Shoulder blade movement and stability Head and cervical placement
Dead bug	Breathing control Ribcage placement Shoulder blade movement and stability Head and cervical placement
Tabletop toe tapping	Ribcage placement Shoulder blade movement and stability Head and cervical placement
Single leg stretch	Breathing control Ribcage placement Shoulder blade movement and stability Head and cervical placement
Scissors	Breathing control Ribcage placement Shoulder blade movement and stability Head and cervical placement
Side leg: lower and lift	Breathing control Pelvic and ribcage placement Shoulder blade movement and stability Head and cervical placement
Side leg: kick	Breathing control Pelvic placement
Side leg: circle	Pelvic placement
Side leg: bicycle	Pelvic and ribcage placement Shoulder blade movement and stability Head and cervical placement
Shoulder bridge	Breathing control Pelvic and ribcage placement
Knee push-up	Breathing control Pelvic and ribcage placement Shoulder blade movement and stability Head and cervical placement
Swimming prep	Pelvic and ribcage placement
Superman	Shoulder blade movement and stability
Swan	Head and cervical placement

**Table 2:** Changes in general characteristics of control (CON) and mat Pilates (MP) groups at the beginning (pre-test) of the study and after 12 weeks (post-test).

Variables	CON (*n* = 17) (Males/Females: 5/12)		MP (*n* = 17) (Males/Females: 5/12)		Time		Group		Time × Group	
	Pre-Test	Post-Test	Pre-Test	Post-Test	ES	*p*-Value	ES	*p*-Value	ES	*p*-Value
Body mass (kg)	58.06 ± 7.87	57.91 ± 8.35	55.27 ± 8.80	55.04 ± 8.95 #	0.098	0.411	0.599	0.014	0.634	0.010
BMI (kg/m^2^)	22.82 ± 1.84	23.12 ± 2.38	22.07 ± 2.07	22.24 ± 2.72	0.185	0.248	0.418	0.060	0.346	0.095
Waist/hip ratio	0.85 ± 0.05	0.86 ± 0.06	0.86 ± 0.05	0.84 ± 0.05 #	0.124	0.352	0.526	0.027	0.196	0.226
Body fat (%)	29.98 ± 3.88	29.73 ± 4.13	27.42 ± 6.05	25.84 ± 4.57 *#	0.322	0.111	0.578	0.017	0.282	0.141
Body fat (kg)	16.07 ± 2.10	17.08 ± 2.47	15.03 ± 3.59	14.35 ± 3.83 *#	0.349	0.094	0.317	0.114	0.014	0.765
LBM (kg)	38.03 ± 6.89	37.58 ± 7.05	35.09 ± 6.37	42.06 ± 5.88 *	0.617	0.012	0.030	0.656	0.398	0.068

Values are means ± SD. *, *p* < 0.05, significant difference within a group; #, *p* < 0.05, significant difference between groups at the same phase. ES, effect size; BMI, body mass index; LBM, lean body mass.

**Table 3:** Changes in cardiovascular functions at the beginning (pre-test) and after 12 weeks (post-test) in the control (CON) and mat Pilates (MP) groups.

Variables	CON (*n* = 17)		MP (*n* = 17)		Time		Group		Time × Group	
	Pre-Test	Post-Test	Pre-Test	Post-Test	ES	*p*-Value	ES	*p*-Value	ES	*p*-Value
HR (bpm)	77.47 ± 9.26	74.65 ± 9.22	72.76 ± 13.03	71.82 ± 9.97	0.303	0.125	0.127	0.346	0.103	0.400
SBP (mmHg)	145.12 ± 17.23	143.12 ± 17.99	141.12 ± 7.83	127.65 ± 11.85 *#	0.757	0.002	0.578	0.017	0.568	0.019
DBP (mmHg)	82.82 ± 10.87	79.71 ± 7.91	78.53 ± 8.12	70.82 ± 9.86	0.035	0.628	0.138	0.326	0.037	0.621
MABP (mmHg)	103.59 ± 12.12	99.12 ± 9.97	98.02 ± 7.80	91.41 ± 9.05 *#	0.411	0.063	0.346	0.096	0.148	0.307
PP (mmHg)	62.29 ± 11.79	63.41 ± 14.80	64.65 ± 8.94	56.82 ± 9.06 *#	0.761	0.002	0.555	0.021	0.645	0.009
CO (L/min)	5.97 ± 1.81	5.82 ± 1.28	6.08 ± 0.98	6.58 ± 1.02	0.195	0.234	0.103	0.399	0.076	0.472
SV (mL/min)	81.71 ± 24.81	74.88 ± 21.37	80.68 ± 15.37	91.14 ± 15.89 *#	0.001	0.950	0.418	0.060	0.023	0.696
SVR (Dynes·s/cm⁵)	1587.47 ± 812.26	1500.71 ± 372.61	1296.59 ± 306.82	1172.47 ± 198.48 *#	0.139	0.324	0.320	0.112	0.172	0.267
Right baPWV (cm/s)	1677 ± 215.14	1650.41 ± 196.57	1661.88 ± 318.99	1542.76 ± 138.27	0.102	0.401	0.007	0.828	0.004	0.869
Left baPWV (cm/s)	1660 ± 207.88	1645.00 ± 174.36	1666.94 ± 314.87	1542.18 ± 154.71	0.058	0.534	0.003	0.896	0.002	0.910
IMT (mm)	0.63 ± 0.76	0.65 ± 0.83	0.61 ± 0.11	0.57 ± 0.81 #	0.013	0.769	0.541	0.024	0.231	0.190
LF/HF ratio	2.31 ± 2.34	1.61 ± 1.50	1.29 ± 1.12	2.52 ± 1.32 *	0.638	0.01	0.266	0.155	0.249	0.172

Values are means ± SD. HR, heart rate; SBP, systolic blood pressure; DBP, diastolic blood pressure; MABP, mean arterial blood pressure; PP, pulse pressure; SVR, systemic vascular resistance; CO, cardiac output; SV, stroke volume; baPWV, brachial–ankle pulse wave velocity; IMT, intima–media thickness; LF/HF ratio, low frequency-to-high frequency ratio of heart rate variability; *, *p* < 0.05, significant difference within a group; #, *p* < 0.05, significant difference between groups at the same period.

## Data Availability

All data supporting this study will be made available from the corresponding authors upon reasonable request.

## References

[1] Kumma W.P., Lindtjørn B., Loha E. (2021). Prevalence of hypertension, and related factors among adults in Wolaita, southern Ethiopia: A community-based cross-sectional study. PLoS ONE.

[2] Saheera S., Krishnamurthy P. (2020). Cardiovascular Changes Associated with Hypertensive Heart Disease and Aging. Cell Transplant.

[3] Cuspidi C., Tadic M., Grassi G., Mancia G. (2018). Treatment of hypertension: The ESH/ESC guidelines recommendations. Pharmacol. Res..

[4] Lionakis N. (2012). Hypertension in the elderly. World J. Cardiol..

[5] Masodsai K., Lin Y.-Y., Lin S.-Y., Su C.-T., Lee S.-D., Yang A.-L. (2021). Aging Additively Influences Insulin- and Insulin-Like Growth Factor-1-Mediated Endothelial Dysfunction and Antioxidant Deficiency in Spontaneously Hypertensive Rats. Biomedicines.

[6] Dharmashankar K., Widlansky M.E. (2010). Vascular Endothelial Function and Hypertension: Insights and Directions. Curr. Hypertens. Rep..

[7] Hansen L., Taylor W.R. (2016). Is increased arterial stiffness a cause or consequence of atherosclerosis?. Atherosclerosis.

[8] Ambrosino P., Bachetti T., D’anna S.E., Galloway B., Bianco A., D’agnano V., Papa A., Motta A., Perrotta F., Maniscalco M. (2022). Mechanisms and Clinical Implications of Endothelial Dysfunction in Arterial Hypertension. J. Cardiovasc. Dev. Dis..

[9] Benincasa G., Coscioni E., Napoli C. (2022). Cardiovascular risk factors and molecular routes underlying endothelial dysfunction: Novel opportunities for primary prevention. Biochem. Pharmacol..

[10] Hong Y., Yang A.-L., Wong J.K., Masodsai K., Lee S.-D., Lin Y.-Y. (2022). Exercise intervention prevents early aged hypertension-caused cardiac dysfunction through inhibition of cardiac fibrosis. Aging.

[11] Myers J., Kaykha A., George S., Abella J., Zaheer N., Lear S., Yamazaki T., Froelicher V. (2004). Fitness versus physical activity patterns in predicting mortality in men. Am. J. Med..

[12] Cornelissen V.A., Smart N.A. (2013). Exercise training for blood pressure: A systematic review and meta-analysis. J. Am. Heart Assoc..

[13] 13 DiMeo F., Pagonas N., Seibert F., Arndt R., Zidek W., Westhoff T.H. (2012). Aerobic Exercise Reduces Blood Pressure in Resistant Hypertension. Hypertens..

[14] Esmailiyan M., Amerizadeh A., Vahdat S., Ghodsi M., Doewes R.I., Sundram Y. (2021). Effect of Different Types of Aerobic Exercise on Individuals With and Without Hypertension: An Updated Systematic Review. Curr. Probl. Cardiol..

[15] De Sousa E.C., Abrahin O., Ferreira A.L.L., Rodrigues R.P., Alves E.A.C., Vieira R.P. (2017). Resistance training alone reduces systolic and diastolic blood pressure in prehypertensive and hypertensive individuals: Meta-analysis. Hypertens. Res..

[16] He L., Wei W.R., Can Z. (2018). Effects of 12-week brisk walking training on exercise blood pressure in elderly patients with essential hypertension: A pilot study. Clin. Exp. Hypertens..

[17] Guimaraes G.V., Cruz L.G.d.B., Fernandes-Silva M.M., Dorea E.L., Bocchi E.A. (2014). Heated water-based exercise training reduces 24-hour ambulatory blood pressure levels in resistant hypertensive patients: A randomized controlled trial (HEx trial). Int. J. Cardiol..

[18] Jin Y.Z., Yan S., Yuan W.X. (2017). Effect of isometric handgrip training on resting blood pressure in adults: A meta-analysis of randomized controlled trials. J. Sports Med. Phys. Fit..

[19] Inder J.D., Carlson D.J., Dieberg G., McFarlane J.R., Hess N.C., A Smart N. (2015). Isometric exercise training for blood pressure management: A systematic review and meta-analysis to optimize benefit. Hypertens. Res..

[20] Edwards J.J., Deenmamode A.H.P., Griffiths M., Arnold O., Cooper N.J., Wiles J.D., O’Driscoll J.M. (2023). Exercise training and resting blood pressure: A large-scale pairwise and network meta-analysis of randomised controlled trials. Br. J. Sports Med..

[21] Gambardella J., Morelli M.B., Wang X., Santulli G. (2020). Pathophysiological mechanisms underlying the beneficial effects of physical activity in hypertension. J. Clin. Hypertens..

[22] Roque F.R., Briones A.M., García-Redondo A.B., Galán M., Martínez-Revelles S., Avendaño M.S., Cachofeiro V., Fernandes T., Vassallo D.V., Oliveira E.M. (2013). Aerobic exercise reduces oxidative stress and improves vascular changes of small mesenteric and coronary arteries in hypertension. Br. J. Pharmacol..

[23] Boeno F.P., Ramis T.R., Munhoz S.V., Farinha J.B., Moritz C.E., Leal-Menezes R., Ribeiro J.L., Christou D.D., Reischak-Oliveira A. (2020). Effect of aerobic and resistance exercise training on inflammation, endothelial function and ambulatory blood pressure in middle-aged hypertensive patients. J. Hypertens..

[24] Roychowdhury D. (2020). Using Physical Activity to Enhance Health Outcomes Across the Life Span. J. Funct. Morphol. Kinesiol..

[25] Lopes S., Félix G., Mesquita-Bastos J., Figueiredo D., Oliveira J., Ribeiro F. (2021). Determinants of exercise adherence and maintenance among patients with hypertension: A narrative review. Rev. Cardiovasc. Med..

[26] Casonatto J., Yamacita C.M. (2019). Pilates exercise and postural balance in older adults: A systematic review and meta-analysis of randomized controlled trials. Complement. Ther. Med..

[27] Pinto J.R., Santos C.S., Soares W.J.S., Ramos A.P.S., Scoz R.D., de Júdice A.F.T., Ferreira L.M.A., Mendes J.J.B., Amorim C.F. (2022). Is pilates better than other exercises at increasing muscle strength? A systematic review. Heliyon.

[28] Hyun A.-H., Cho J.-Y., Koo J.-H. (2022). Effect of Home-Based Tele-Pilates Intervention on Pregnant Women: A Pilot Study. Healthcare.

[29] Lim E.-J., Hyun E.-J. (2021). The Impacts of Pilates and Yoga on Health-Promoting Behaviors and Subjective Health Status. Int. J. Environ. Res. Public Health.

[30] Zaras N., Kavvoura A., Gerolemou S., Hadjicharalambous M. (2023). Pilates-mat training and detraining: Effects on body composition and physical fitness in pilates-trained women. J. Bodyw. Mov. Ther..

[31] Carrasco-Poyatos M., Ramos-Campo D.J., Rubio-Arias J.A. (2019). Pilates versus resistance training on trunk strength and balance adaptations in older women: A randomized controlled trial. PeerJ.

[32] da Luz M.A., Costa L.O.P., Fuhro F.F., Manzoni A.C.T., Oliveira N.T.B., Cabral C.M.N. (2014). Effectiveness of Mat Pilates or Equipment-Based Pilates Exercises in Patients With Chronic Nonspecific Low Back Pain: A Randomized Controlled Trial. Phys. Ther..

[33] Almeida I.d.S., Andrade L.d.S., de Sousa A.M.M., Junior G.C., Catai A.M., Mota Y.L., Durigan J.L.Q. (2022). Is the Combination of Aerobic Exercise with Mat Pilates Better than Mat Pilates Training Alone on Autonomic Modulation Related to Functional Outcomes in Hypertensive Women? Secondary Analysis of a Randomized Controlled Trial. Int. J. Environ. Res. Public Health.

[34] Fourie M., Gildenhuys G., Shaw I., Shaw B., Toriola A., Goon D. (2013). Effects of a Mat Pilates Programme on Body Composition in Elderly Women. West Indian Med. J..

[35] González-Devesa D., Varela S., Diz-Gómez J.C., Ayán-Pérez C. (2024). The efficacy of Pilates method in patients with hypertension: Systematic review and meta-analysis. J. Hum. Hypertens..

[36] Martins-Meneses D.T., Antunes H.K.M., de Oliveira N.R.C., Medeiros A. (2014). Mat Pilates training reduced clinical and ambulatory blood pressure in hypertensive women using antihypertensive medications. Int. J. Cardiol..

[37] Rocha J., Cunha F.A., Cordeiro R., Monteiro W., Pescatello L.S., Farinatti P. (2020). Acute Effect of a Single Session of Pilates on Blood Pressure and Cardiac Autonomic Control in Middle-Aged Adults With Hypertension. J. Strength Cond. Res..

[38] Tanaka H., Monahan K.D., Seals D.R. (2000). Age-predicted maximal heart rate revisited. Circ..

[39] Kyle U.G., Bosaeus I., De Lorenzo A.D., Deurenberg P., Elia M., Gomez J.M., Heitmann B.L., Kent-Smith L., Melchior J.-C., Pirlich M. (2004). Bioelectrical impedance analysis? Part I: Review of principles and methods. Clin. Nutr..

[40] Masodsai K., Chaunchaiyakul R. Determination of Cardiac Function Using Impedance Cardiography During Jogging With and Without Breast Support. Enhancing Health and Sports Performance by Design. MoHE 2019. Lecture Notes in Bioengineering.

[41] Gambassi B.B., Neves V.R., Brito E.Z.A., Fernandes D.S.D.S., Sá C.A., Nogueira R.M.D.R., Almeida F.D.J.F., Cavalcanti P.A.D.A., E Silva D.C.G.G., Neto D.S. (2020). A validation study of a smartphone application for heart rate variability assessment in asymptomatic adults. Am. J. Cardiovasc. Dis..

[42] Tedla Y.G., Bautista L.E. (2015). Drug Side Effect Symptoms and Adherence to Antihypertensive Medication. Am. J. Hypertens..

[43] Hellsten Y., Nyberg M. (2015). Cardiovascular Adaptations to Exercise Training. Compr Physiol..

[44] Gomez-Cabrera M.-C., Domenech E., Viña J. (2008). Moderate exercise is an antioxidant: Upregulation of antioxidant genes by training. Free. Radic. Biol. Med..

[45] Mann N., Rosenzweig A. (2012). Can Exercise Teach Us How to Treat Heart Disease?. Circulation.

[46] Pal S., Radavelli-Bagatini S., Ho S. (2013). Potential benefits of exercise on blood pressure and vascular function. J. Am. Soc. Hypertens..

[47] Makki K., Froguel P., Wolowczuk I. (2013). Adipose tissue in obesity-related inflammation and insulin resistance: Cells, cytokines, and chemokines. ISRN Inflamm..

[48] Tanaka T., Narazaki M., Kishimoto T. (2014). IL-6 in Inflammation, Immunity, and Disease. Cold Spring Harb. Perspect. Biol..

[49] Tsiotra P., Tsigos C., Raptis S. (2001). TNFα and leptin inhibit basal and glucose-stimulated insulin secretion and gene transcription in the HIT-T15 pancreatic cells. Int. J. Obes..

[50] Jiménez-Maldonado A., Montero S., Lemus M., Cerna-Cortés J., Rodríguez-Hernández A., Mendoza M.A., Melnikov V., Gamboa-Domínguez A., Muñiz J., Virgen-Ortiz A. (2019). Moderate and high intensity chronic exercise reduces plasma tumor necrosis factor alpha and increases the Langerhans islet area in healthy rats. J. Musculoskelet. Neuronal Interact..

[51] Talebi-Garakani E., Safarzade A. (2012). Resistance training decreases serum inflammatory markers in diabetic rats. Endocrine.

[52] Ito Y., Nomura S., Ueda H., Sakurai T., Kizaki T., Ohno H., Izawa T. (2002). Exercise training increases membrane bound form of tumor necrosis factor-α receptors with decreases in the secretion of soluble forms of receptors in rat adipocytes. Life Sci..

[53] Seals D.R., Nagy E.E., Moreau K.L. (2019). Aerobic exercise training and vascular function with ageing in healthy men and women. J. Physiol..

[54] Pedralli M.L., Waclawovsky G., Camacho A., Markoski M.M., Castro I., Lehnen A.M. (2016). Study of endothelial function response to exercise training in hypertensive individuals (SEFRET): Study protocol for a randomized controlled trial. Trials.

[55] Touyz R.M. (2004). Reactive Oxygen Species, Vascular Oxidative Stress, and Redox Signaling in Hypertension. Hypertension.

[56] Fukai T., Folz R.J., Landmesser U., Harrison D.G. (2002). Extracellular superoxide dismutase and cardiovascular disease. Cardiovasc. Res..

[57] Graham D.A., Rush J.W.E. (2004). Exercise training improves aortic endothelium-dependent vasorelaxation and determinants of nitric oxide bioavailability in spontaneously hypertensive rats. J. Appl. Physiol..

[58] Higashi Y., Sasaki S., Kurisu S., Yoshimizu A., Sasaki N., Matsuura H., Kajiyama G., Oshima T. (1999). Regular aerobic exercise augments endothelium-dependent vascular relaxation in normotensive as well as hypertensive subjects: Role of endothelium-derived nitric oxide. Circulation.

[59] Goto C., Higashi Y., Kimura M., Noma K., Hara K., Nakagawa K., Kawamura M., Chayama K., Yoshizumi M., Nara I. (2003). Effect of Different Intensities of Exercise on Endothelium-Dependent Vasodilation in Humans: Role of Endothelium-Dependent Nitric Oxide and Oxidative Stress. Circulation.

[60] Wang X., Wang Z., Tang D. (2021). Aerobic Exercise Alleviates Inflammation, Oxidative Stress, and Apoptosis in Mice with Chronic Obstructive Pulmonary Disease. Int. J. Chronic Obstr. Pulm. Dis..

[61] Takabe W., Jen N., Ai L., Hamilton R., Wang S., Holmes K., Dharbandi F., Khalsa B., Bressler S., Barr M.L. (2011). Oscillatory Shear Stress Induces Mitochondrial Superoxide Production: Implication of NADPH Oxidase and c-Jun NH_2_-Terminal Kinase Signaling. Antioxidants Redox Signal..

[62] Fujii J., Homma T., Osaki T. (2022). Superoxide Radicals in the Execution of Cell Death. Antioxidants.

[63] Ye Y., Lin H., Wan M., Qiu P., Xia R., He J., Tao J., Chen L., Zheng G. (2021). The Effects of Aerobic Exercise on Oxidative Stress in Older Adults: A Systematic Review and Meta-Analysis. Front. Physiol..

